# The Prospective SPOTLESS Trial: Setup Accuracy of Tattoo-Less Surface-Guided Breast Radiotherapy Including Regional Nodal Irradiation

**DOI:** 10.3390/jcm15166253

**Published:** 2026-08-13

**Authors:** Eva Meixner, Sophia Albert, Bahar Cepni, David Neugebauer, Hin Hoi Lau, Julian Thater, Klaus Herfarth, Stephan Mende, Fabian Weykamp, Adriana Ayestaran-Aldaz, Line Hoeltgen, Nathalie Arians, Jakob Liermann, Lars Wessel, Semi Harrabi, Hanna Waldsperger, Jürgen Debus, Sebastian Klüter, Vania Batista

**Affiliations:** 1 Department of Radiation Oncology, Heidelberg University Hospital, Im Neuenheimer Feld 400, 69120 Heidelberg, Germany; sophia.albert@med.uni-heidelberg.de (S.A.); bahar.cepni@med.uni-heidelberg.de (B.C.); hin.lau@med.uni-heidelberg.de (H.H.L.); julian.thater@med.uni-heidelberg.de (J.T.); klaus.herfarth@med.uni-heidelberg.de (K.H.); stephanmatthias.mende@med.uni-heidelberg.de (S.M.); fabian.weykamp@med.uni-heidelberg.de (F.W.); adriana.ayestaranaldaz@med.uni-heidelberg.de (A.A.-A.); line.hoeltgen@med.uni-heidelberg.de (L.H.); nathalie.arians@med.uni-heidelberg.de (N.A.); jakob.liermann@med.uni-heidelberg.de (J.L.); lars.wessel@med.uni-heidelberg.de (L.W.); semi.harrabi@med.uni-heidelberg.de (S.H.); hanna.waldsperger@med.uni-heidelberg.de (H.W.); juergen.debus@med.uni-heidelberg.de (J.D.); sebastian.klueter@med.uni-heidelberg.de (S.K.); vania.batista@med.uni-heidelberg.de (V.B.); 2 Heidelberg Institute of Radiation Oncology (HIRO), 69120 Heidelberg, Germany; 3 National Center for Tumor Diseases (NCT), 69120 Heidelberg, Germany; 4 Heidelberg Ion Therapy Center (HIT), Im Neuenheimer Feld 450, 69120 Heidelberg, Germany; 5 German Cancer Research Center (DKFZ), Clinical Cooperation Unit Radiation Oncology, Im Neuenheimer Feld 280, 69120 Heidelberg, Germany

**Keywords:** VMAT, DIBH, breast cancer

## Abstract

**Background/Objectives**: The implementation of tattoo-free radiotherapy (RT) for breast cancer reflects an effort to mitigate the psychological distress of permanent marks through surface-guided techniques, while simultaneously evaluating its utility in meeting setup precision requirements. **Methods**: In this prospective trial, patients were positioned for breast cancer RT including regional nodal irradiation exclusively using surface-guided RT (SGRT). Cone-beam computed tomography (CBCT) was acquired at each fraction as the ground truth to analyze translational setup deviations. **Results**: A total of 457 paired SGRT–CBCT data points in 30 patients were acquired. A residual translational setup deviation of ≤6 mm was achieved in 97.6% of all fractions with a median deviation of 2 mm (range: 0–12). Neither age, comorbidities, body mass index, extent of target volumes, choice of breathing technique, nor patient-reported symptoms (pain, dermatitis, anxiety) correlated significantly with setup deviations. The median in-room positioning time was 92 s (range: 20 s–6 min and 37 s) and significantly prolonged in patients with an elevated BMI and higher grades of pain and skin dermatitis, and for chest wall irradiation. Manual assessment of the accuracy of regional nodal CTVs in each CBCT relative to the planning CT showed good-to-minor deviations in 99.7% (Level 1/2), 95.0% (Supra-/infraclavicular), and 90.8% (Internal mammary), respectively, with major deviations in only 0.3% (Level 1/2), 5.0% (Supra-/infraclavicular) and 9.2% (Internal mammary). A forward dose calculation on CBCT geometries (*n* = 86) revealed high median CTV dose coverage of 100.0% (range: 57.1–107.7%) of the original target dose for all lymph node levels combined. **Conclusions**: The tattoo-free positioning setup for regional nodal irradiation in breast cancer patients exhibited pronounced robustness, maintaining its accuracy and reliability irrespective of patient-, tumor-, or treatment-specific variables with high clinical efficiency. However, larger cohorts are required to confirm whether these parameters genuinely operate independently of setup accuracy.

## 1. Introduction

Permanent skin tattoos have long been the standard for patient positioning in breast cancer radiotherapy (RT), serving as reliable, inexpensive, and technically simple landmarks [1]. However, these marks are increasingly recognized as a source of adverse cosmetic and psychological effects, often serving as a permanent reminder of the disease [2]. In an international survey, approximately 70% of women viewed tattoos negatively, and 78% expressed a preference for tattoo-free treatment despite potential additional costs or time [3]. Concerns regarding body image and self-esteem are particularly relevant for breast cancer survivors, for whom treatment completion is a key milestone in recovery.

Advanced surface-guided radiotherapy (SGRT) systems provide a non-invasive alternative by using optical surface imaging for real-time, three-dimensional monitoring. This technology utilizes high-resolution cameras to generate a digital map of the patient’s skin, which is compared to a reference mesh derived from the planning CT [4]. By constantly calculating deviations, SGRT enables precise alignment and continuous motion tracking without additional radiation dose. This approach is particularly advantageous for regions subject to motion, such as the breast, and can reduce the frequency of CBCT imaging, thereby lowering overall radiation exposure [5,6,7].

Several studies have demonstrated comparable, and in some cases superior setup accuracy compared with conventional tattoo-based workflows in accelerated partial breast irradiation setups [8,9] or whole breast RT [10,11,12,13,14].

Shifting toward tattoo-free methods aligns with patient-centered care by enhancing comfort and satisfaction. However, data on setup accuracy for breast cancer patients requiring regional nodal irradiation remain limited. The prospective SPOTLESS study (Setup accuracy of tattoo-less surface-guided breast radiotherapy with regional nodal irradiation) aims to evaluate real-world setup accuracy using the AlignRT^®^ system in patients receiving adjuvant breast or chest wall radiotherapy including regional lymph node irradiation.

## 2. Materials and Methods

### 2.1. Study Design and Objectives

The SPOTLESS (setup accuracy of tattoo-less surface-guided breast radiotherapy with regional nodal irradiation) trial was conducted as a prospective, single-center phase II trial including 30 breast cancer patients scheduled for adjuvant radiotherapy to the breast or chest wall including regional lymph nodes. The analysis was approved by the local ethical review board (S-647/2025) and performed in accordance with the ethical standards laid down in the 1964 Declaration of Helsinki and its later amendments. The study was registered at DRKS on 14 January 2026 (DRKS00038990).

Patients fulfilling the following criteria were considered: indication for image-guided, postoperative photon radiotherapy of the breast or chest wall including regional nodal irradiation; ECOG Performance status ≤ 2; ability to understand the trial; written informed consent; and age ≥ 18 years. Exclusion criteria include pregnancy, lactation, or comorbidities/anatomical reasons preventing standard positioning in the Wingstep^®^ immobilization device.

### 2.2. Study Procedures

Patient positioning and the planning CT scan were performed in the supine position with arms raised in the Wingstep^®^ device (IT-V, Innsbruck, Austria) and a slice thickness of 3 mm. All patients were positioned using the tattoo-less AlignRT^®^ (VisionRT Ltd., London, UK) surface-guided radiotherapy system for initial setup without permanent skin markings. Surface-guided RT was used to generate a 3D scan. For RT planning, contouring of clinical target volumes (CTV) of the breast or chest wall and regional nodal levels (axilla level 1–2, axilla level 3/infraclavicular region, level 4/supraclavicular region, interpectoral nodes, and the internal mammary nodes region) was performed by deep learning segmentation in Raystation treatment planning system (TPS) and manually corrected by board-certified radiation oncologists according to ESTRO guidelines [15]. A planning target volume (PTV) was generated, encompassing a 5–7 mm margin around all CTVs. Treatment was planned as moderately hypofractionated, volumetric modulated arc radiotherapy with photons in 15–16 fractions with a single dose of 2.5 to 2.67 Gy with or without a simultaneously integrated boost using free-breathing or deep inspiration breath hold techniques.

Positioning verification: Patients were positioned based on surface guidance using the AlignRT system to achieve a setup deviation ≤ 3 mm and translational 3° per institutional standard. A reference image was acquired during the first fraction to facilitate positioning for subsequent sessions. The setup time, defined from the start to the completion of SGRT positioning for each fraction, was recorded. Immediately after SGRT-based positioning, CBCT (cone-beam computed tomography) was acquired to verify setup accuracy and record deviations. Only translational displacements (vertical, longitudinal, lateral) were allowed for the registration process for both online guidance and offline analysis. The registration should focus on a close match of the target volume and bony anatomy, minimizing deviations and averaging them when they arise.

### 2.3. Manual and Dosimetric Assessment

The visual accuracy of the regional nodal CTVs in each CBCT scan relative to the planning CT was manually graded by one blinded observer using a four-tier classification system based on the severity of deviations:

1. Good alignment: Characterized by optimal geometric coverage with no relevant deviations.
2. Minor deviations: Mild misalignment of the CTV that do not compromise the geometric or clinical validity.
3. Major deviations: Moderate to severe misalignments of the CTV requiring close monitoring or optimization, yet remaining within a clinically tolerable threshold.
4. Unacceptable deviations: Critical misalignment defined as any instance where the CTV is no longer fully enclosed within the PTV.

For a dosimetric assessment, the CBCT scans of the first, sixth and eleventh fractions for each patient were imported into RayStation TPS (version RS2024B) and co-registered with the original planning CT using rigid image registration without rotational correction. An algorithm implemented in the TPS was used to generate a corrected CBCT (cCBCT). The algorithm converted the CBCT intensity to CT-like Hounsfield units and supplemented regions outside the field of view (FoV) using information from the planning CT, while preserving the anatomical information within the CBCT FoV. Regional nodal CTV levels were re-contoured on each CBCT. Finally, for forward dose computation, the isocenter was transferred using the rigid image registration, and the original treatment plan was then recalculated on the corrected CBCT using the clinical dose engine to enable subsequent dose–volume histogram evaluation. Target coverage was evaluated using D95% (the dose delivered to 95% of the Clinical Target Volume, expressed as a percentage of the prescribed target dose).

### 2.4. Patient-Reported Outcomes (PROs)

Patient comfort and RT-related adverse events were assessed using numerical rating scale questionnaires of PRO-CTCAE (Item Library Version 1.0 [16]) at the planning CT, start of RT, and end of RT. The questionnaire evaluated domains including general well-being, body image, anxiety, and adverse events with pain, dyspnea, fatigue, and skin dermatitis. To account for fluctuations across the three assessment points, the maximum score recorded for each patient was utilized for the analysis.

### 2.5. Statistical Analysis

The sample size of 30 patients, resulting in approximately 450–480 paired SGRT–CBCT data points, was based on the primary endpoint and published data on SGRT accuracy [10,11,17]. The primary objective was to evaluate the real-world accuracy of tattoo-free patient positioning in breast cancer radiotherapy including regional nodal irradiation. Technical practicability for an acceptable setup accuracy was concluded if ≥90% of treatment fractions meet the predefined criteria of residual setup deviation ≤ 6 mm in all translational directions following surface-guided alignment, verified by daily CBCT. Secondary objectives included the assessment of treatment workflow efficiency (e.g., setup time) and factors, which could potentially affect patient positioning, and the reproducibility of positioning accuracy.

Descriptive statistics were calculated for setup deviations. All enrolled patients receiving at least one fraction were included in the intention-to-treat analysis. Data collection included patient-, tumor-, and therapy-related characteristics such as age, body mass index, TNM classification, and target volumes. A *p*-value < 0.05 was defined as statistically significant. Comparative analysis using chi-square, Fisher’s exact test, and logistic regression was performed to adjust for confounders. Statistical analysis was performed using statistical software SPSS (IBM Corp., Armonk, NY, USA, version 27.0).

### 2.6. Dropout and Missing Data

Four patients dropped out for the following reasons: inability to achieve the required arm positioning with the Wingstep immobilization device (*n* = 1), omission of regional nodal irradiation (*n* = 1), subsequent withdrawal of patient consent (*n* = 1), and cancellation of the RT (*n* = 1).

Five fractions in one patient were excluded because the immobilization setup was applied incorrectly at planning CT. Following a re-planning-CT scan, the remaining 10 fractions for the patient were included. One patient discontinued RT after 15 of the planned 16 fractions for personal reasons (stay abroad). To ensure optimal target matching, four fractions (0.9%) required manual correction of the automated matching by a board-certified radiation oncologist.

## 3. Results

Thirty-four patients were initially enrolled in this study, of whom 4 dropped out prior to planning CT scan, leaving a total of 30 patients with 457 paired SGRT–CBCT data points available for the final analysis. The baseline characteristics of the final study cohort are summarized in Table 1 and Table 2.

### 3.1. Setup Accuracy and Positioning Time

A total of 457 paired SGRT–CBCT data points were available. Technical practicability, defined as a residual setup deviation of ≤6 mm in all translational directions, was achieved in 97.6% (*n* = 446, 95 CI: 95.7–98.8%) of all treatment fractions. Only 11 (2.4%) fractions in eleven patients exhibited a setup deviation exceeding 6 mm; thus, the primary endpoint of the study was successfully achieved. Overall, a median deviation in all translational directions of 2 mm (range: 0–12 mm) was assessed. The median longitudinal, lateral, and vertical deviations were 2 mm (range: 0–12 mm) in superior/inferior direction, 2 mm (range: 0–9 mm) in right/left direction, and 2 mm (range: 0–10 mm) in anterior/posterior direction.

The median in-room setup time, defined from the start to the completion of SGRT positioning was recorded in 443 of 457 fractions (96.9%) and was 92 s (range: 20 s to 6 min and 37 s).

### 3.2. Analysis of Influencing Factors

The impact of patient- and therapy-related characteristics on the probability of meeting the primary accuracy endpoint with a residual deviation ≤ 6 mm is summarized below:

Patient age (*p* = 0.553), ECOG performance score (*p* = 0.216) or Charlson comorbidity index (*p* = 0.743) did not show a significant correlation with setup accuracy. No significant differences were observed regarding either absolute BMI (*p* = 0.112) or the presence of overweight (BMI ≥ 25 kg/m^2^) (*p* = 0.346) with the change in all translational deviations.

No significant differences in positioning deviations were observed among patients treated for intact breasts, chest wall or implants (*p* = 0.226). Furthermore, neither the CTV (*p* = 0.307), the PTV (*p* = 0.372) nor the laterality (*p* = 0.156) was a significant confounder on the reproducibility of the Wingstep^®^ immobilization and SGRT alignment. The choice of breathing technique (DIBH vs. FB) had no significant impact on translational shifts, neither overall (*p* = 0.361) nor when analyzed individually for the superior/inferior (*p* = 0.696), right/left (*p* = 0.475), and anterior/posterior (*p* = 0.107) directions.

Patient-reported outcomes—including general health status (*p* = 0.159), body satisfaction (*p* = 0.721), and radiation-related anxiety (*p* = 0.603)—showed no significant correlation with the primary endpoint. Likewise, radiation-related adverse events, such as fatigue (*p* = 0.137), dyspnea (*p* = 0.431), skin dermatitis (*p* = 0.973), and pain (*p* = 0.501), did not significantly correlate with an increased rate of CBCT deviations.

A significantly prolonged median in-room positioning time was observed in patients with an elevated BMI (*p* = 0.020), for patients with chest wall irradiation (*p* = 0.010) compared to intact breast or implant, and for patients with higher grades of pain (*p* = 0.034) and skin dermatitis (*p* = 0.038).

### 3.3. Manual and Dosimetric Assessment

The manual assessment of visual accuracy of regional nodal CTVs was performed in all 457 CBCTs compared to the planning CT. The extent of regional nodal irradiation varied among the cohort, with not all patients undergoing treatment to every lymph node region. Specific details of the target volumes are provided in Table 1. The manual assessment demonstrated predominantly favorable results for the level 1 + 2 lymph node levels, whereas infra- and supraclavicular and mammary interna regions exhibited greater variability:

Level 1/2 (*n* = 334 fractions): Showed good-to-minor deviations in 99.7% (*n* = 333) of cases, with major deviations observed in 0.3% (*n* = 1). Level 3/4 (*n* = 442 fractions): Demonstrated good-to-minor deviations in 95.0% (*n* = 420) of cases, while major deviations were present in 5.0% (*n* = 22). Internal mammary region (*n* = 426 fractions): Exhibited good-to-minor deviations in 90.8% (*n* = 387) of cases, with major deviations noted in 9.2% (*n* = 39). Unacceptable deviations, defined as CTV no longer fully enclosed within the PTV, did not occur in any of the regional nodal levels.

**Figure 1:**
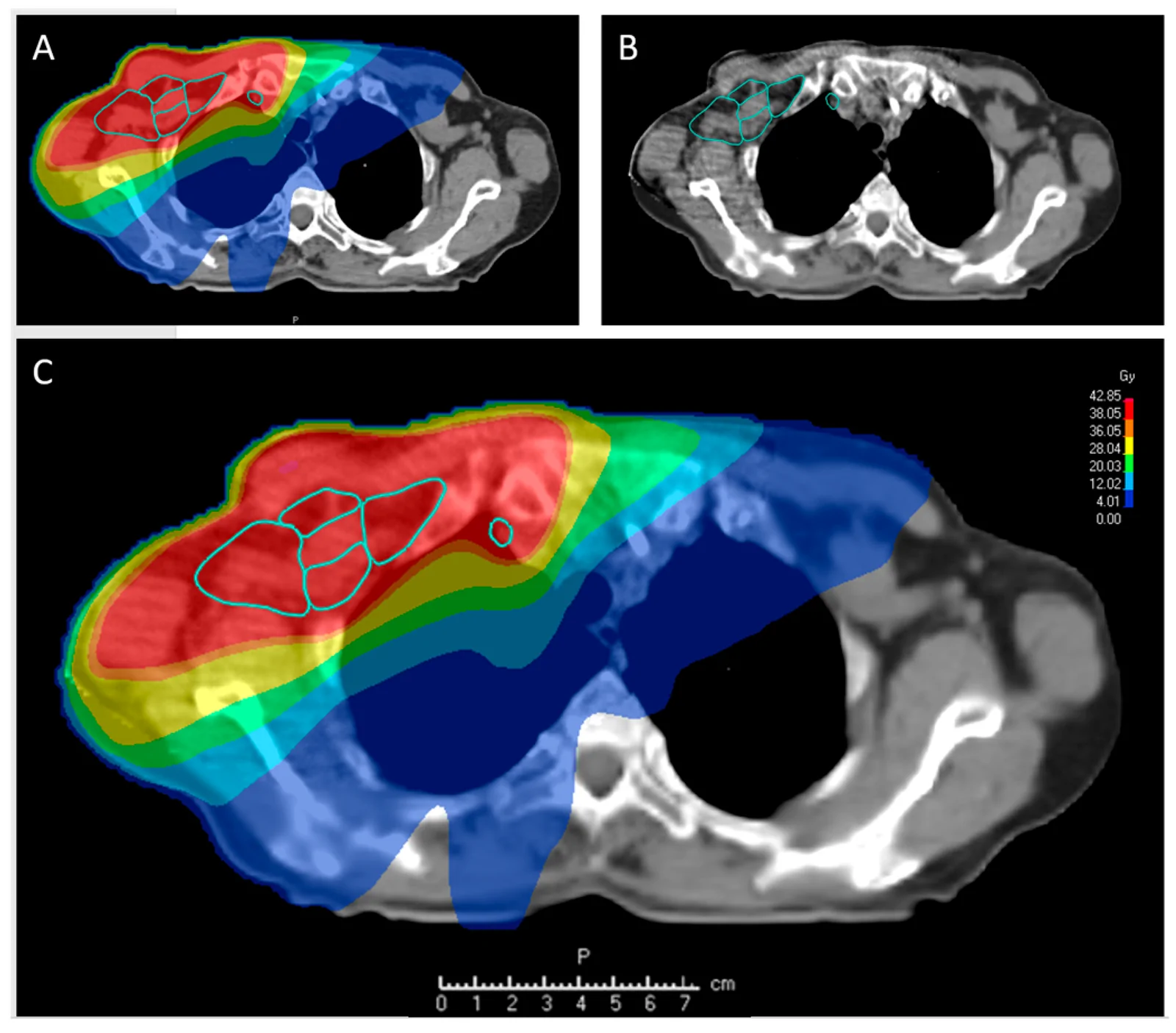
Forward calculation for a patient with right-sided regional nodal irradiation. Planning-CT and treatment plan (**A**), rigidly registered cone-beam computer tomography (**B**), and forward calculation (**C**).

The forward dose calculation of the original treatment plans onto the fused cCBCT geometries was performed for a total number of 86 fractions (Figure 1, median of 3 CBCTs for each patient; range: 2–4) and revealed the following median CTV dose coverages relative to the prescribed CTV dose: The median CTV dose coverage encompassed 100.0% of the original target dose (range: 57.1–107.7%) for all lymph node levels combined. Table 3 provides a detailed overview of the coverage for each individual lymph node level. The minimum coverage value of 57.1% for the internal mammary CTV was observed in a single fraction for one patient, whereas overall coverage across all remaining fractions remained clinically adequate.

### 3.4. Patient-Reported Outcomes (PROs)

The results from the PRO-CTCAE questionnaires at the three time points (planning CT, start of RT, end of RT) are detailed below. In terms of general health, 13 (43.3%) participants reported a very good to rather good status, while 11 (36.7%) rated it as moderate and 6 (20%) as poor. Similarly, body satisfaction was assessed as rather good (*n* = 11, 36.7%), moderate (*n* = 13, 43.3%), or rather poor (*n* = 6, 20%). Assessment of patient apprehension towards RT revealed that 73.3% (*n* = 22) experienced none/mild anxiety, 13.3% (*n* = 4) reported moderate, and 13.3% (*n* = 4) strong/very strong anxiety.

Radiation-induced symptoms with fatigue were reported as none to mild in 9 (30%), moderate in 9 (30%), and severe in 12 (40%) patients. The occurrence of dyspnea was graded as follows: none to mild in 21 (70%), moderate in 6 (20%) and severe in 3 patients (10%). RT-associated pain was reported as none to mild in 26 (86.7%), moderate in 1 (3.3%) and severe in 3 (10%) patients, while RT-related skin dermatitis was graded as: none to mild in 21 (70%), moderate in 4 (13.3%) and severe in 5 (16.7%) patients.

## 4. Discussion

This prospective study demonstrates that tattoo-less surface-guided RT for breast cancer patients receiving regional nodal irradiation is clinically feasible, highly accurate, and eliminates the need for permanent skin markings. Our findings indicate that using surface topography for initial setup and real-time motion management provides robust spatial positioning, irrespective of patient-, tumor-, or treatment-specific variables. Crucially, this high level of precision was maintained even when treating complex regional lymph node volumes, which inherently demand strict immobilization and accurate multi-axis alignment.

Our data demonstrates that the median translational setup errors remained well within clinically acceptable margins, matching the accuracy profiles within errors of 2–6 mm reported by previous studies that further found SGRT comparable or superior to traditional tattoo-based markers for whole breast RT [8,10,17,18]. Kügele et al. [10] reported a superior patient setup with SGRT for whole breast RT compared to a tattoo-based laser setup with a median vector offset of 4.2 mm for the laser-based technique compared to 2.4 mm for SGRT. Rudat et al. [17] reported a mean translational setup error of 3.6 mm for SGRT compared to 4.5 mm using laser alignment with skin tattoos. In addition to optimizing the initial setup accuracy for SGRT compared to the traditional three-point laser-based tattoo setup method, the work of Hattel et al. [18] highlights another critical advantage of SGRT: its capacity for continuous, real-time, intrafractional motion monitoring and quantification as a quality assurance tool throughout the entire treatment fraction. Their study demonstrates that intrafractional motion remained remarkably minimal, averaging approximately 1.1 mm from the isocenter. Furthermore, the robustness of an intrafractional SGRT setup approach is supported by the extensive dataset analyzed by Reitz et al. [19]. Across a total of 2028 treatment RT sessions of the whole breast or chest wall with a mean treatment session time of 154 s, intra-fractional motion remained remarkably well-controlled with a median change of 1.63 mm during dose application.

Our findings demonstrate that the tattoo-free setup approach is highly robust against patient-specific, therapy-related, and tumor-specific factors. In our cohort, variations in age, comorbidities, body mass index, target volumes and breathing technique did not significantly impair the setup precision or target coverage. This high procedural robustness aligns with a retrospective study of Giantsoudi et al. [5] that found no significant correlation of BMI or breath hold technique to accuracy of SGRT setup. Such stability is fundamentally expected from SGRT. Unlike traditional, point-based laser alignment with permanent tattoos—which is inherently prone to skin shift, geometric variations, and localized anatomical distortion—SGRT leverages dense, three-dimensional surface topologies. By utilizing thousands of virtual reference points over a broad region of interest, the system inherently dampens localized noise and mitigates confounding patient or therapy variables.

Although the study of Giantsoudi et al. [5] included patients undergoing regional nodal irradiation, a detailed sub-analysis evaluating the setup accuracy specifically for individual lymph node levels was not performed. A key finding of our study is the comprehensive anatomical and dosimetric analysis of the individual lymph node levels. Despite this overall high accuracy for all lymph node levels, our data revealed that the coverage and setup accuracy were lowest for the internal mammary chain (IMC) compared to the other nodal stations. Several factors might have contributed to this phenomenon. The small volume of the IMC CTV inherently increased its susceptibility to anatomical variations, rotational errors, and a matching workflow that compromises on highly localized, paramedian areas like the IMC in favor of an optimized global fit. For the IMC, even minor geometric shifts or chest wall excursion deviations could lead to disproportionate volumetric or dosimetric impacts. However, dosimetric forward calculation revealed a clinically acceptable dose coverage. Our observations align with the previous literature emphasizing that while SGRT provides excellent overall target localization, the tight spatial corridor of the IMC—bordering critical organs at risk like the heart and internal mammary vessels—remains the most sensitive to residual interfractional and intrafractional motion as well as dose coverage in RT planning [20,21].

A central question remains whether SGRT can eventually replace daily 2D-kV or 3D-CBCT image guidance. While SGRT excels at detecting external surface deviations and rotations, it cannot visualize internal soft tissue or nodal levels directly. In this study, all patients received daily CBCT for verifying the position of regional lymph nodes and assessing the influence of internal anatomical changes. While 2D kV/Portal imaging may suffice for bone-based alignment for whole breast RT, the high precision required for regional nodal irradiation suggests that SGRT should be viewed as a complement to, rather than a total replacement for, 3D internal verification CBCT to ensure the safest possible dose delivery to complex target volumes. As the SGRT workflow showed marked improvements over laser-based tattoo setup, a reduction in daily imaging for patient setup might be possible [22]. Further studies and clinical trials are necessary to validate the required and optimal imaging frequency protocol for comprehensive nodal workflows.

Several limitations of the present study must be acknowledged. First, the lack of a randomized controlled design restricts our ability to directly compare the tattoo-free workflow with traditional setups in a strictly controlled manner, which may introduce selection bias. Additionally, all patients were immobilized exclusively using a Wingstep device. While this standardizes our internal data, it remains unclear whether the high accuracy and resistance to anatomical factors observed here would translate to other common setups, such as vacuum cushions, boards without arm supports, or prone positioning. Another limitation of this study is that multiple observations per patient were analyzed without adjusting for intra-individual clustering, as statistical tests assuming independence were applied. Consequently, within-patient dependencies were not fully accounted for. A limitation of this study is the sample size (*n* = 30), which was insufficient to formally rule out subtle or moderate associations between setup accuracy and variables such as BMI, breathing technique, or target volume. The absence of statistically significant differences across these subgroups should be interpreted with caution, as non-significance does not demonstrate equivalence or the definitive absence of an effect. Larger, prospective multi-center studies are warranted to confirm these subgroup findings. Additionally, as a single-center study, the institutional workflows and specific technology configurations (such as translational correction only) may reflect local practices that might vary across other radiation oncology departments. Furthermore, while we extensively analyzed daily setup accuracy, we did not account for continuous intrafractional motion tracking or detailed post-treatment verification scans. Finally, all gradings were performed by a single blinded observer. Although this ensured consistent scoring across all data, it precludes the evaluation of inter-observer reliability.

Despite these limitations, a major strength of this study lies in its rigorous prospective design, which involved an intensive analysis of every individual lymph node level across all daily CBCTs, thereby significantly expanding the current literature on SGRT in complex regional nodal irradiation for breast cancer.

## 5. Conclusions

The present findings demonstrate that the tattoo-free surface-guided radiotherapy workflow for irradiation of breast cancer patients including regional nodal irradiation exhibited pronounced robustness, maintaining its accuracy and reliability irrespective of patient-, tumor-, or treatment-specific variables and could be executed with high clinical efficiency and throughput. However, larger cohorts are required to confirm whether these parameters genuinely operate independently of setup accuracy.

## Figures and Tables

**Table 1:** Patient and treatment characteristics.

Characteristics	Value (Percentage or Range)
**Median age**; years	52 (24–76)
**Median BMI;** kg/m^2^	23.9 (18.6–37.6)
**Median ECOG**	0 (0–1)
**Median CCI**	3 (2–5)
**Laterality**	
Right	16 (53.3%)
Left	14 (46.6%)
**Target volume**	
chest wall	10 (33.3%)
breast	12 (40.0%)
implant	8 (26.7%)
**Regional nodal irradiation**	
Lv. 1–2	1 (3.3%)
Lv. 1–4	1 (3.3%)
Lv. 3–4, internal mammary region	9 (30.0%)
Lv. 1–4, internal mammary region	19 (63.3%)
**Fractions**	
15 fractions	17 (56.7%)
16 fractions	13 (43.3%)
**Breathing technique**	
Free-breathing	8 (26.7%)
Deep inspiration breath hold	22 (73.3%)
**Simultaneously integrated tumor bed boost**	
Yes	16 (53.3%)
No	14 (46.7%)

BMI: body mass index, CCI: Charlson Comorbidity Index, ECOG: Eastern Cooperative Oncology Group Performance Status, Lv: Level.

**Table 2:** Clinical and planning target volume characteristics.

Characteristics	Value (Range)
**Median target CTV**; cm^3^	641 (155–1690)
**Median PTV**; cm^3^	1136 (505–2350)
Median PTV chest wall; cm^3^	792 (505–1350)
Median PTV breast; cm^3^	1278 (895–2350)
Median PTV implant; cm^3^	1062 (553–1576)
**Median regional nodal irradiation CTV**; cm^3^	127 (29–255)
Median CTV Lv. 1; cm^3^	77 (38–122)
Median CTV Lv. 2; cm^3^	19 (8–42)
Median CTV Lv. 3; cm^3^	14 (8–20)
Median CTV Lv. 4; cm^3^	16 (8–25)
Median CTV interpectoral; cm^3^	10 (4–22)
Median CTV internal mammary node; cm^3^	10 (7–15)

CTV: clinical target volume, Lv: Level, PTV: planning target volume.

**Table 3:** Dosimetric analysis with forward calculation.

Regional Nodal Irradiation	CTV Coverage on cCBCT Percentage (Range)
Level 1 (*n* = 60)	100.2 (97.9–102.6)%
Level 2 (*n* = 60)	100.3 (79.8–101.5)%
Level 3 (*n* = 83)	100.0 (83.0–103.0)%
Level 4 (*n* = 83)	100.0 (75.3–107.7)%
Internal mammary node (*n* = 80)	99.6 (57.1–105.6)%
Interpectoral (*n* = 86)	100.0 (93.4–104.7)%

cCBCT: corrected cone-beam computed tomography, CTV: clinical target volume.

## Data Availability

The raw data supporting the conclusions of this article will be made available by the authors on request insofar as it complies with local ethics committee regulations.

## Abbreviations

The following abbreviations are used in this manuscript:

BMI	Body mass index
CBCT	Cone-beam computed tomography
cCBCT	Corrected cone-beam computed tomography
CCI	Charlson Comorbidity Index
CT	Computed tomography
CTV	Clinical Target Volume
DIBH	Deep Inspiration breath hold
ECOG	Eastern Cooperative Oncology Group Performance Status
FoV	Field of view
Lv	Level
PRO	Patient-Reported Outcomes
PTV	Planning target volume
RT	Radiotherapy
SGRT	Surface-guided RT
TPS	Treatment planning system
